# Supplementary material for: The tale of springs and streams: how different aquatic ecosystems impacted the mtDNA population structure of two riffle beetles in the Western Carpathians
Source: PeerJ. 2020 Oct 6;8:e10039. doi: 10.7717/peerj.10039 (PMC7546224; doi:10.7717/peerj.10039)
Supplement: Supplemental Information 1 — Map of (A) the studied area within the Carpathian Arc and (B) the 73 sampling sites (36 springs and 37 streams) divided into seven river basins represented by different color fill.The grey line represents the state borders of Slovakia (SK), Hungary (H), Ukraine (UA), Poland (PL), Czech Republic (CZ) and Austria (AU). [file peerj-08-10039-s001.pdf]

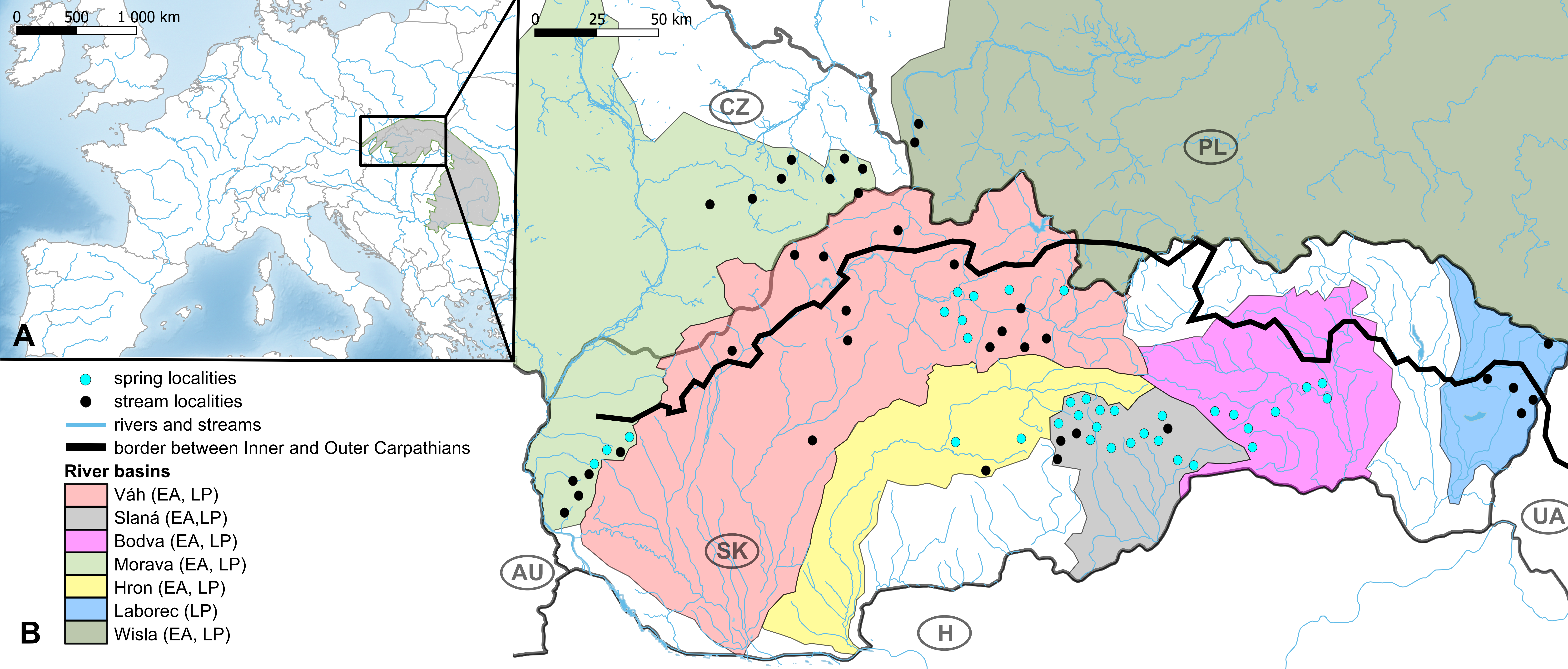

0
500
1 000 km
A
0
25
50 km
CZ
PL
SK
AU
H
UA
spring localities
stream localities
rivers and streams
border between Inner and Outer Carpathians
River basins
Váh (EA, LP)
Slaná (EA,LP)
Bodva (EA, LP)
Morava (EA, LP)
Hron (EA, LP)
Laborec (LP)
Wisla (EA, LP)
B
