# Supplementary figures and images for: The tale of springs and streams: how different aquatic ecosystems impacted the mtDNA population structure of two riffle beetles in the Western Carpathians
Source: PeerJ. 2020 Oct 6;8:e10039. doi: 10.7717/peerj.10039 (PMC7546224; doi:10.7717/peerj.10039)

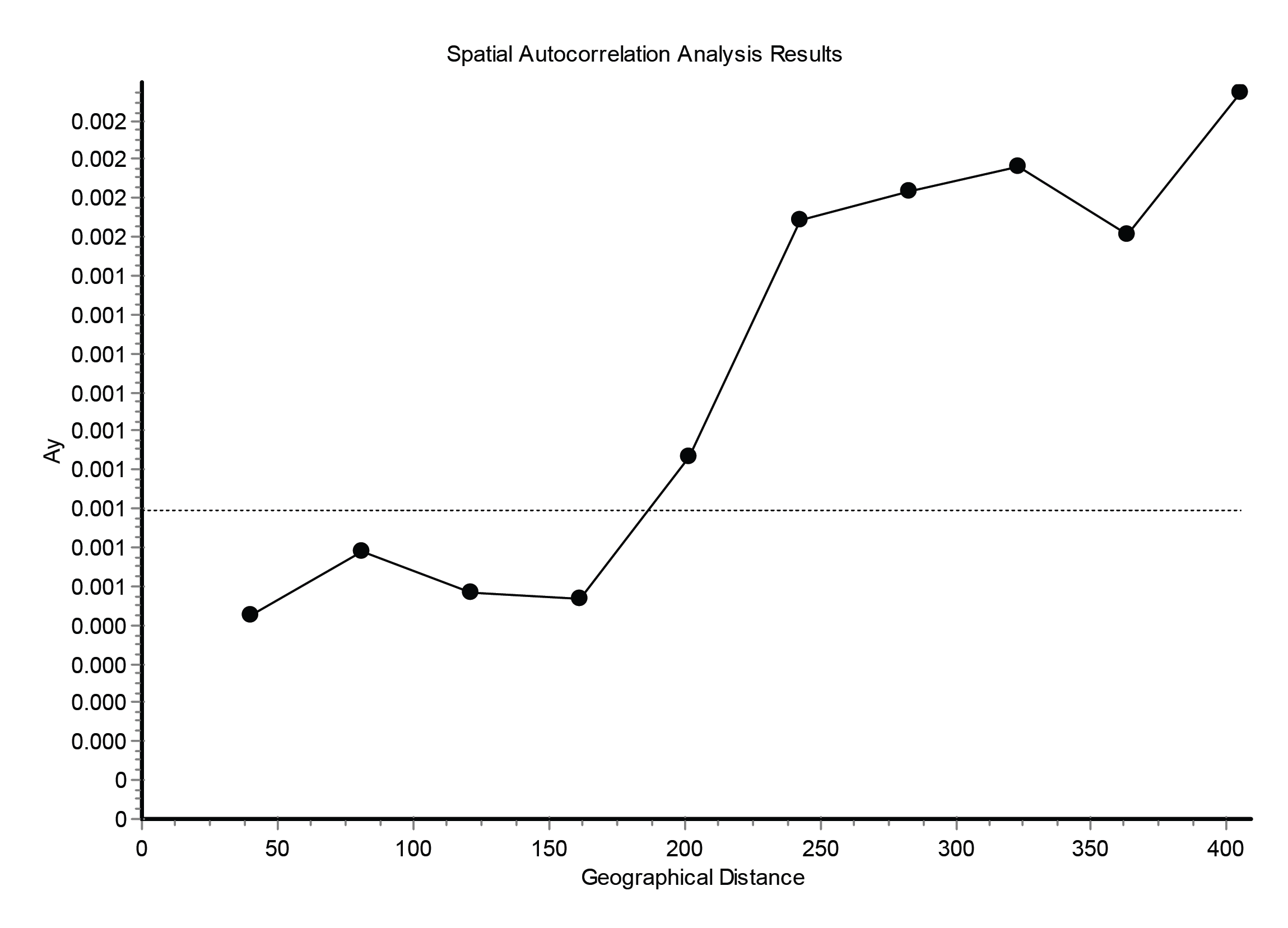

Supplement: Supplemental Information 2 — Distance classes (10) are given on the x-axis between 0 and 413 km, and pairwise genetic distances (Ay) are given on the y-axis.The horizontal dotted line indicates the average genetic distance for the observed data set. Values above the horizontal line suggest a correlation between distance class and genetic distance. [file peerj-08-10039-s002.png]
