# Supplementary material for: The tale of springs and streams: how different aquatic ecosystems impacted the mtDNA population structure of two riffle beetles in the Western Carpathians
Source: PeerJ. 2020 Oct 6;8:e10039. doi: 10.7717/peerj.10039 (PMC7546224; doi:10.7717/peerj.10039)
Supplement: Supplemental Information 3 [file peerj-08-10039-s003.docx]

| **Mts System^1^** | **Geomorphological Unit** | **Geomorphological Subunit** | **Code** | **Locality** | **Habitat^2^** | **River basin** | **GPS Coordinates** | **Altitude** | **Species^3^** |
| --- | --- | --- | --- | --- | --- | --- | --- | --- | --- |
| **IWC** | **FTA**  Fatra-Tatra Area  (Fatránsko-tatranská oblasť) | **FTA1**  Little Carpathians (Malé Karpaty) | V006 | Stužková | SP | Morava | 48.595833, 17.453567 | 250 m | EA (3) |
|  |  |  | V009 | Chrenkech jarok | SP | Morava | 48.654283, 17.619650 | 328 m | EA (10) |
|  |  |  | V070 | Orešanská | SP | Morava | 48.451800, 17.346483 | 324 m | EA (1), LP (3) |
|  |  |  | KCH3 | Kuchyňa | ST | Morava | 48.403491, 17.154763 | 241 m | EA (7) |
|  |  |  | LIB1 | Libuša 1 | ST | Morava | 48.500500, 17.324700 | 374 m | LP (9) |
|  |  |  | LIM1 | Limbašský 1 | ST | Morava | 48.294700, 17.175000 | 342 m | LP (3) |
|  |  |  | LOZ4 | Lozorno 4 | ST | Morava | 48.324600, 17.090000 | 265 m | LP (1) |
|  |  |  | SPV1 | Stupavský 1 | ST | Morava | 48.252500, 17.112217 | 292 m | EA (7), LP (8) |
|  |  | **FTA2**  High Tatras (Vysoké Tatry) | BEL3 | Belá | ST | Váh | 49.113900, 19.834700 | 809 m | LP (9) |
|  |  | **FTA3**  Low Tatras (Nízke Tatry) | V020 | Bukovinka I | SP | Váh | 49.003350, 19.282567 | 653 m | EA (7) |
|  |  |  | V022 | Bukovinka III | SP | Váh | 49.003300, 19.285083 | 631 m | EA (6) |
|  |  |  | BOC2 | Boca 2 | ST | Váh | 48.996700, 19.763200 | 697 m | LP (5) |
|  |  |  | BYS4 | Nižné Bystré | ST | Váh | 48.944000, 19.632800 | 1638 m | LP (4) |
|  |  |  | HOD1 | Hodruša 1 | ST | Váh | 48.960300, 19.826000 | 877 m | LP (9) |
|  |  |  | SVA1 | Svarinka 1 | ST | Váh | 48.959900, 19.891900 | 983 m | LP (8) |
|  |  | **FTA4**  Western Tatras (Západné Tatry)  Váh river basin | V027 | Prosiek 2 | SP | Váh | 49.157917, 19.497483 | 642 m | EA (2) |
|  |  |  | V028 | Prosiek 3 | SP | Váh | 49.157383, 19.498019 | 639 m | EA (3) |
|  |  |  | V086 | Liptovská Anna | SP | Váh | 49.159783, 19.462033 | 845 m | EA (2), LP (4) |
|  |  | **FTA5**  Great Fatra (Veľká Fatra | V033 | Jazierce | SP | Váh | 49.018200, 19.281900 | 589 m | EA (6) |
|  |  | **FTA6**  Belianske Tatras (Belianske Tatry) | V036 | Dolina 7 prameňov | SP | Váh | 49.222800, 20.277600 | 1208 m | LP (1) |
|  |  | **FTA7**  Strážov Mts (Strážovské vrchy) | NIT1 | Nitra 1 | ST | Váh | 48.657484, 18.637691 | 671 m | EA (4), LP (1) |
|  |  |  | SVI1 | Svinianka 1 | ST | Váh | 49.149437, 18.650388 | 456 m | EA (1) |
|  |  |  | TUR1 | Turiec 1 | ST | Váh | 48.964780, 18.727228 | 575 m | EA (9), LP (4) |
|  | **SCM**  Slovak Central Mts  (Slovenské stredohorie) | **SCM1**  Podpoľanie | V087 | Oravická | SP | Hron | 48.700667, 19.272938 | 394 m | EA (6) |
|  |  | **SCM2**  Poľana Mts (Poľana) | KAM1 | Kamenistý | ST | Hron | 48.662400, 19.628500 | 884 m | LP (9) |
|  | **SOM**  Slovak Ore Mts  (Slovenské rudohorie) | **SOM1**  Slovak Karst (Slovenský kras) | V037 | Prameň sv. Jána | SP | Bodva | 48.653850, 20.974667 | 264 m | EA (6) |
|  |  |  | V038 | Drieňovská | SP | Bodva | 48.607583, 20.964650 | 187 m | EA (11) |
|  |  |  | V042 | Fej | SP | Bodva | 48.609367, 20.749017 | 222 m | EA (10) |
|  |  |  | V043 | Tapolča I | SP | Bodva | 48.583600, 20.686883 | 198 m | EA (12) |
|  |  |  | V044 | Tapolča II | SP | Bodva | 48.584050, 20.688838 | 204 m | EA (6) |
|  |  |  | V045 | Eveteš | SP | Bodva | 48.598633, 20.643438 | 255 m | EA (6) |
|  |  |  | V046 | Čierna | SP | Slaná | 48.562683, 20.465317 | 248 m | EA (7) |
|  |  |  | V047 | Biela | SP | Slaná | 48.567583, 20.468050 | 237 m | EA (6) |
|  |  |  | V048 | Kečovská | SP | Slaná | 48.500100, 20.485817 | 331 m | EA (6) |
|  |  |  | V049 | Krásnohorská | SP | Slaná | 48.617600, 20.587233 | 336 m | EA (9) |
|  |  |  | V050 | Brzotínska | SP | Slaná | 48.608783, 20.470933 | 247 m | EA (6) |
|  |  |  | V051 | Vidová | SP | Slaná | 48.564317, 20.440167 | 238 m | EA (6) |
|  |  |  | V052 | pod Vápenkou | SP | Slaná | 48.554933, 20.419170 | 212 m | EA (6) |
|  |  |  | V073 | Studený | SP | Slaná | 48.571583, 20.400850 | 224 m | EA (5) |
|  |  |  | V075 | Hučiaca B | SP | Slaná | 48.625167, 20.389900 | 269 m | EA (6) |
|  |  |  | V088 | Drieňov kúpele | SP | Bodva | 48.624500, 20.952000 | 257 m | EA (6), LP (3) |
|  |  | **SOM2**  Muráň Plateau (Muránska planina) | V057 | Brusík | SP | Slaná | 48.831400, 20.010100 | 574 m | LP (3) |
|  |  |  | V058 | pod Javorníčkovou | SP | Slaná | 48.724100, 20.013800 | 413 m | LP (1) |
|  |  |  | V060 | Havraník lúka | SP | Hron | 48.813400, 20.071400 | 768 m | LP (5) |
|  |  |  | V061 | Havraník les | SP | Hron | 48.813300, 20.071700 | 766 m | LP (1) |
|  |  |  | V062 | Jelšavská teplica | SP | Slaná | 48.605017, 20.295092 | 255 m | EA (13) |
|  |  |  | V065 | Tisovec | SP | Slaná | 48.692317, 19.967417 | 576 m | EA (1) |
|  |  |  | V066 | Rejkovský | SP | Slaná | 48.668283, 19.925367 | 400 m | EA (6) |
|  |  |  | V067 | Teplice - Furmanec | SP | Slaná | 48.688833, 19.898817 | 476 m | EA (5) |
|  |  |  | V074 | Kunova teplica | SP | Slaná | 48.607333, 20.390933 | 248 m | EA (12) |
|  |  |  | HAV1 | Havraník 1 | ST | Hron | 48.824000, 20.071600 | 761 m | LP (8) |
|  |  |  | HDZ1 | Hrdzavý 1 | ST | Slaná | 48.768200, 19.986800 | 868 m | LP (9) |
|  |  | **SOM3**  Volovec Mts (Volovské vrchy) | SMO1 | Smolník 1 | ST | Laborec | 48.709000, 20.700700 | 635 m | LP (8) |
| **OWC** | **SMC**  Slovak-Moravian Carpathians  (Slovensko-moravské Karpaty) | **SMC1**  Maple Mts (Javorníky) | VAH1 | Váh 1 | ST | Váh | 49.325346, 18.511067 | 582 m | EA (1), LP (7) |
|  |  |  | VYD1 | Vydrňanka | ST | Váh | 49.217800, 18.252780 | 523 m | EA (1), LP (1) |
|  |  | **SMC2**  White Carpathians (Biele Karpaty) | SEL1 | Selecký 1 | ST | Váh | 48.777800, 17.998700 | 374 m | LP (8) |
|  | **WB**  Western Beskids  (Západné Beskydy) | **WB1**  Moravian-Silesian Beskids  (Moravsko-sliezske Beskydy) | CZ01 | Lomná | ST | Morava | 49.547710, 18.650423 | 538 m | EA (9), LP (8) |
|  |  |  | CZ02 | Příslopský | ST | Morava | 49.624213, 18.575444 | 497 m | EA (2), LP (6) |
|  |  |  | CZ03 | Satina | ST | Morava | 49.565317, 18.422775 | 772 m | EA (4), LP (10) |
|  |  |  | CZ04 | Černa Ostravice | ST | Morava | 49.456600, 18.470900 | 816 m | LP (3) |
|  |  |  | CZ05 | Kněhyně | ST | Morava | 49.462546, 18.278190 | 570 m | EA (6), LP (9) |
|  |  |  | CZ06 | Malá Bystřička | ST | Morava | 49.394775, 18.053709 | 456 m | EA (7), LP (1) |
|  |  |  | CZ07 | Bystřička | ST | Morava | 49.371673, 17.750556 | 563 m | EA (6), LP (4) |
|  |  |  | KYS1 | Kysuca 1 | ST | Váh | 49.431857, 18.626540 | 570 m | EA (8), LP (9) |
|  |  | **WB2**  Orava Magura (Oravská Magura) | BRE1 | Brezovica 1 | ST | Váh | 49.343800, 19.662100 | 687 m | LP (6) |
|  |  | **WB3**  Silesian Beskids  (Sliezske Beskydy) | PL04 | Żyłica | ST | Wisla | 49.693800, 18.984000 | 609 m | LP (8) |
|  |  |  | PL05 | Labajów | ST | Wisla | 49.622821, 18.869191 | 523 m | EA (3), LP (7) |
|  | **CB**  Central Beskids  (Stredné Beskydy) | **CB1**  Kysucké Beskydy | OSC1 | Oščadnica | ST | Váh | 49.421200, 18.910810 | 822 m | EA (3), LP (6) |
| **IEC** | **VM**  Vihorlat Mts  (Vihorlatské vrchy) | **VM1**  Vihorlat Mts (Vihorlatské vrchy) | BAR1 | Barnov 1 | ST | Laborec | 48.938400, 22.160300 | 434 m | LP (9) |
|  |  |  | HRA1 | Hrabový 1 | ST | Laborec | 48.878000, 22.297500 | 412 m | LP (7) |
|  |  |  | KRV1 | Krivec 1 | ST | Laborec | 48.907300, 22.203700 | 569 m | LP (5) |
|  |  |  | ROV1 | Rovný 1 | ST | Laborec | 48.887200, 22.324200 | 311 m | LP (7) |
| **OEC** | **PM**  Poloniny Mts (Poloniny) | **PM1**  Poloniny Mts (Poloniny) | ZBJ2 | Zbojský | ST | Laborec | 49.050500, 22.513700 | 773 m | LP (5) |

**Mts System^1^: IWC** Inner Western Carpathians, **OWC** Outer Western Carpathians, **IEC** Inner Eastern Carpathians, **OEC** Outer Eastern Carpathians**; Habitat^2^: SP** spring, **ST** stream**; Species^3^: EA** *Elmis aenea***, LP** *Limnius perrisi* (numbers in parentheses indicate the number of individuals for each locality)
