# Supplementary material for: The tale of springs and streams: how different aquatic ecosystems impacted the mtDNA population structure of two riffle beetles in the Western Carpathians
Source: PeerJ. 2020 Oct 6;8:e10039. doi: 10.7717/peerj.10039 (PMC7546224; doi:10.7717/peerj.10039)
Supplement: Supplemental Information 4 — The subpopulation is defined as individuals of one locality within the drainage basins, Table S1, Fig. S1). [file peerj-08-10039-s004.docx]

| ***E. aenea*** | |  |  |  |  |  |  |
| --- | --- | --- | --- | --- | --- | --- | --- |
| **Source of variation** | | **df^1^** | **SS^2^** | **Variance components** | **% of variation** | **F value** | **p-value** |
| Among subunits | | 5 | 2.862 | -0.00141 | - 0.70 | F_CT_ = -0.007 | > 0.323 |
| Among subpopulations within subunits | | 41 | 24.237 | 0.08122 | 40.39 | F_SC_ = 0.401 | > 0.000 |
| Within subpopulations | | 229 | 27.774 | 0.12128 | 60.31 | F_ST_ = 0.396 | < 0.000 |
| ***L. perrisi*** |  |  | |  |  |  |  |
| **Source of variation** | | **df^1^** | **SS^2^** | **Variance components** | **% of variation** | **F value** | **p-value** |
| Among subunits | | 6 | 0.441 | 0.00011 | 0.25 | F_CT_ = 0.0025 | > 0.121 |
| Among subpopulations within subunits | | 36 | 2.364 | 0.00456 | 10.03 | F_SC_ = 0.101 | > 0.092 |
| Within subpopulations | | 199 | 8.117 | 0.04079 | 89.72 | F_ST_ = 0.103 | < 0.075 |

**df^1^** Degree of freedom, **SS^2^** Sum of squares
